# What Are the Priorities in Malaria Research?

**DOI:** 10.1371/journal.pmed.0030083

**Published:** 2006-01-31

## Abstract

The Multilateral Initiative on Malaria (MIM) has held its fourth conference, during which current key issues for research were discussed. Lessons can be learned from MIM's success in boosting malaria research.

Malaria remains a challenging prospect for researchers and health workers, but there is encouraging news to report. Malaria research, after many years on the back burner, has risen dramatically up the priority list of donors and policy makers. Much of the credit for this turnaround must go to the Multilateral Initiative on Malaria (MIM; http://www.mim.su.se). MIM's achievements in the malaria world may indeed be a model for raising the profile of other neglected health issues.

MIM defines itself as “an alliance of organizations and individuals concerned with malaria.” It aims to maximize the impact of research on malaria in Africa, through promoting capacity building and facilitating global collaboration and coordination. Its key areas of focus include: optimizing the use of current drugs and developing new ones, capacity building in Africa, overcoming obstacles to vaccine development, malaria control and management, vector biology and control, the economics of malaria, health information systems and surveillance, and research methodology. MIMCom ( http://www.mimcom.net) has provided Internet connectivity at 20 research sites in Africa, and created some useful Internet resources. Other malaria initiatives have been launched in the past 10 years, and MIM has worked in partnership with many of them.

The first meeting of MIM was in Dakar, Senegal, in 1997. Fewer than 200 people were present. In marked contrast, the fourth conference, held in Yaoundé, Cameroon, in November of last year, was attended by more than 1,500 participants from nearly 70 countries. An encouraging number were young, enthusiastic, and African, one of whom won the Young Malaria Scientist of the Year award. The recipient, Genevieve Fouda Amou'ou, a Cameroonian working at Georgetown University in Washington, DC, United States, won the award for her work on the characterization of the malarial antibody response of Cameroonian infants. There were 600 posters and 200 oral presentations at the meeting (see Box 1 for crucial issues addressed). The conference also heard news of another welcome development: the MIM secretariat will be moving from Sweden to an African base, in Dar es Salaam—a clear reaffirmation of the organization's commitment to building capacity inside Africa itself. Impressive global media coverage of the event was achieved by the MIM press team, further raising the profile of malaria internationally. Our policy at *PLoS Medicine* is to focus on diseases with the greatest global burden, and we have published many papers on malaria since our launch (see http://medicine.plosjournals.org/perlserv/?request=get-static&name=collection-malaria). The expansion of Internet access for malaria researchers has made open-access online publication an effective means of sharing the results of research. It is, regrettably, unlikely that researchers in other key health areas working in remote locations in Africa have anything like this level of connectivity. MIMCom could usefully share its experience with those working in other research fields.

What did delegates make of their conference? Opinions varied on the quality of the research presented, and there were many who felt that the sessions describing current progress with international malaria programs merely presented information that was readily available elsewhere—for example, on the Web sites of the organizations concerned. Nevertheless, the networking opportunities afforded by the conference were universally appreciated. Sadly, there are many African malaria scientists who, for financial or logistical reasons, were unable to attend the conference; travel within Africa is difficult, and delegates from countries quite close to Cameroon found themselves obliged to fly via Europe. But for those who made it, this was an event that, though frequently hectic, provided a boost for morale.

It was, however, an event primarily for researchers. Only a minority of those present were involved in the treatment of malaria. As always, getting research into practice presents its own challenges.

**Box 1. Fourth MIM Pan-African Malaria Conference: The Crucial Issues**

- Getting artemisinin-based combination therapy (ACT) to as many people as possible.
- Finding the next generation of drugs, to be used when resistance to ACT inevitably appears.
- Investigating further the promising concept of intermittent treatment in childhood and pregnancy.
- Getting as many people as possible to use insecticide-treated bed nets.
- Improving other aspects of vector control: repellents, residual sprays, and larviciding.
- The RTS,S/AS02A malaria vaccine candidate has shown promise in early trials (Lancet 364: 1411–1420). Can it be made into an affordable, safe, and effective vaccine that is quickly available to all those who need it?
- There are 24 other candidate vaccines awaiting clinical trials. More genomic research will produce further candidates, but some doubt that this is how malaria resources are best spent; they argue that it is more important to trial the candidates we already have.
- It is increasingly recognized that the nature of the malaria problem varies from place to place. Regional and local research initiatives are needed to develop appropriate policies.
- Malaria is (or at least should be) addressed by several interventions at once, but research usually evaluates one intervention at a time. Research is needed to evaluate combined programs.
- There is a pressing need for more research to find the best ways of delivering effective interventions.

## Abbreviation

MIM: Multilateral Initiative on Malaria

